# Variation in the Primary and Secondary Metabolites, Antioxidant and Antibacterial Potentials of Tomatoes, Grown in Soil Blended with Different Concentration of Fly Ash

**DOI:** 10.3390/plants11040551

**Published:** 2022-02-19

**Authors:** Sajid Dominic, Abdullah Ijaz Hussain, Muhammad Hamzah Saleem, Huda Alshaya, Basit Latief Jan, Shafaqat Ali, Xiukang Wang

**Affiliations:** 1 Department of Applied Chemistry, Government College University Faisalabad, Faisalabad 38000, Pakistan; sajid.sipra26@gmail.com; 2 Natural Product and Synthetic Chemistry Lab, Department of Chemistry, Government College University Faisalabad, Faisalabad 38000, Pakistan; 3 College of Plant Science and Technology, Huazhong Agricultural University, Wuhan 430070, China; saleemhamza312@webmail.hzau.edu.cn; 4 Cell and Molecular Biology, University of Arkansas, Fayetteville, NC 72701, USA; hmalshay@uark.edu; 5 Department of Clinical Pharmacy, College of Pharmacy, King Saud University, Riyadh 11451, Saudi Arabia; basitlatief@gmail.com; 6 Department of Environmental Sciences and Engineering, Government College University, Faisalabad 38000, Pakistan; 7 Department of Biological Sciences and Technology, China Medical University, Taichung 40402, Taiwan; 8 College of Life Sciences, Yan’an University, Yan’an 716000, China; wangxiukang@yau.edu.cn

**Keywords:** *Lycopersicon esculentum*, antioxidant, fly ash, antibacterial activity, amino acid

## Abstract

The aim of the study was to investigate the variation in nutritional composition, antioxidant, and antibacterial activities of tomatoes grown in fly ash blended soils, i.e.,T1 (soil 100% + 0% fly ash), T2 (soil 75%+ 25% fly ash), and T3 (soil 50% + 50% fly ash) soils. The tomato samples were harvested and screened for their proximate analysis, mineral composition, vitamin C contents, phenolic profile, and antioxidant and antibacterial activities. Results showed that T3 soil crop has high proximate composition, vitamin C, and phenolic contents as compared with T2 and T1 soil crops. Furthermore, significant variation in the elements analysis was observed in the crops grown in T1, T2, and T3 soils. T3 soil crop exhibited better antioxidant activity in term of total phenolic contents (TPC), total flavonoids contents (TFC), DPPH radical scavenging capacity, and ferric reducing potential as compared with T2 and T1 soil crops. Similarly, maximum inhibition zones were observed for T3 soil crop when tested for *Staphylococcus aureus* isolate 1 and 3 and methicillin-resistant *Staphylococcus aureus* (MRSA). In conclusion, the crop grown in T3 blended soil had high antioxidant and antibacterial potentials and better nutritional composition.

## 1. Introduction

During the combustion of coal, the most cognizant industrial byproduct produced is fly ash [1]. Estimated production of fly ash throughout the world is about 600 million tons per year [2]. The highest production of fly ash from the carbonization of coal, about 2 million tons annually, was observed at the Lakhra coal power plant in Pakistan [3]. Major uses of this fly ash include its use in dam construction, building material, in agriculture as soil amendment, etc. [4]. Across the globe, disposal and management of fly ash is considered a serious problem due to high cost [4]. It also has some adverse effects such as release of some dangerous effluents directly in the biosphere, contaminating ground, surface water, etc. [5]. Besides the adverse effects, fly ash can be used for positive purposes and after mixing with soil, it can show some healthy outcomes on plants growth and increase their nutritional level [6].

There are some reports in literature on the effect of fly ash blended soil on the nutritional composition of *Cajanuscajan*, *Helianthus annus*, *Vigna radiate*, *Solenum melongata*, *Jatrophacurcas*, and *S. oleracea* crops [7]. Low concentration of fly ash amendment in soil increases availability of nutrients and biomass but high concentration of fly ash amendment leads to enhancement of the antioxidant enzymes present [1]. Amendment of optimum concentration of fly ash with soil improves its ameliorated properties such as chemical, biological, and physical characteristics [8]. Optimum amendment of fly ash increases concentration of micro- and macronutrients such as K, Mg, Ca, Na, and Fe that increase its biomass and yield [9]. Other than these essential nutrients, presence of heavy metals such as Se, Cd, Ni, B, Pb, Mo, and Ni accumulate in plants and can cause different issues regarding human consumption [10]. Devastating effects of heavy metals present in fly ash on the environment and growth of plants cannot be ignored. Consequently, urgent need has arisen for the use of fly ash to check variation in nutritional composition, mineral profile, phenolic profile, antioxidant activity, and in vitro antibacterial potential of fruit extracts.

Tomato (*Lycopersicum esculentum* L.) is a basic vegetable that is grown and consumed commercially worldwide due to having nutritional importance [11]. Tomatoes belong to the genus *Lycopersicum*, which is in the same family, *Solanaceae*, as potatoes [12]. Tomato contains high concentration of polyphenols that have potential to act as antioxidant and free radical scavenging. Polyphenols are reported for effects against many degenerative diseases such as cancer, arthritis, and cardiovascular diseases [13,14]. Phenolics are the main polyphenol present in plants and have many biological applications, used in therapeutics, cosmetics, and food industries as ancestors for bioactive molecules [15]. Other than polyphenols, tomato also contains many other valuable nutrients such as vitamin C, minerals, and other vitamins [16]. These nutritional and nutraceutical compounds not only decrease oxidative stress but also protect against fetal disease by reducing the levels of free radicals [17,18]. Thus, the current task was designed to explore the variation in nutritional composition, vitamin C, phenolic profile, mineral profile, antioxidant, and antimicrobial potentials of extracts of tomato fruits growing in soil after blending of different concentrations of fly ash, such as T1 (soil 100% + 0% fly ash), T2 (soil 75% + 25% fly ash), and T3 (soil 50% + 50% fly ash).

## 2. Materials and Methods

### 2.1. Sample Collection

Local tomato plants were grown in three type of soils, blended with fly ash (T1 (soil 100% + 0% fly ash), T2 (soil 75% + 25% fly ash), and T3 (soil 50% + 50% fly ash) soils) in the experimental field of Botanical Garden, Government College University Faisalabad, Pakistan. Fruit samples were collected and packed in polythene bags and transferred to the Natural Product and Synthetic Chemistry (NPSC) Lab, Department of Chemistry, Government College University Faisalabad Pakistan for further analysis.

### 2.2. Reference Compounds, Reagents, and Chemicals

Standards and reference chemicals used in the study, i.e., vitamin C, linoleic acid, Folin–Ciocalteu reagent, 2,2-diphenyl-1-picrylhydrazyl radical (DPPH•), butylated hydroxytoluene (BHT), gallic acid, chlorogenic acid, p-hydroxy benzoic acid, sinapic acid, ferulic acid, vanillic acid, caffeic acid, p-coumeric acid, kaempferol, quercetin, catechin, and rutin, which were procured from Sigma Chemical Co. (St. Louis, MO, USA). All other chemicals, i.e., sulfuric acid, diethyl ether, copper sulfate, sodium sulfate, sodium hydroxide, boric acid, sodium nitrate, aluminum chloride, hydrochloric acid, and methanol of analytical grade used in this study were purchased from Merck (Darmstadt, Germany).

### 2.3. Proximate Analysis

Moisture contents of tomato fruits were observed using the method reported by Osborne and Voogt [19]. The ash contents, crude fat, fiber contents, and crude protein of tomato fruits were measured by using the AOAC methods [20]. Total carbohydrate content was measured as reported by Eyeson and Ankrah [21].

### 2.4. Estimation of Ascorbic Acid

For the estimation of ascorbic acid contents in the tomato fruits, a previously described method was employed [22]. A double beam spectrometer was used for the measurement of absorbance at 515 nm (Spectrophotometer Analytika, Jena, Germany).

### 2.5. Preparation of Extracts

Methanol (MeOH) extracts of tomato fruits were prepared using orbital shaker as reported previously [23]. Briefly, 50 g of air-dried sample was ground with 80 mesh size and soaked in 500 mL absolute methanol and shaken for 24 h using an orbital shaker at 140 rpm. The resulted extracts were concentrated on a rotary vacuum evaporator (BRE-225 Robus Technologies), weighed to calculate the yield, and stored at 4 °C for further analysis.

### 2.6. Evaluation of the Antioxidant Activity of the Extracts

Folin–Ciocalteu phenol reagent was used for the estimation of total phenolic content (TPC) and total flavonoid content (TFC), as mentioned by Hussain et al. [24], and results were reported as mg/100 g of dry weight, measured as gallic acid equivalent (GAE) and catechin equivalent (CE), respectively. The 2,2-diphenyl-1-picrylhydrazyl (DPPH) assay for the measurement of free-radical-scavenging activity of tomato extracts was performed as reported previously [24]. Measurement of the ferric reducing antioxidant activity of tomato extracts was performed by a method suggested by Benzie and Strain [25].

### 2.7. Elemental Detection

Estimation of Na and K was performed using flame photometry (FP-640) and Ca, Cd, Cu, Fe, Mg, Mn, Ni, Pb, and Zn using atomic absorption spectrophotometer (AAS), Perkin Elmer (A. Analyst 300) from Central Hi Tech Lab, Government College University Faisalabad, Pakistan, as reported by Raj et al. [26].

### 2.8. Estimation of Phenolics and Flavonoids by Using HPLC

#### 2.8.1. Sample Hydrolysis

Estimation of phenolics and flavonoids of tomato fruit extracts was performed by a previously reported hydrolysis method [24]. Briefly, precisely one gram of tomato fruit extract was measured and dissolved in 10 mL 50% (*v/v*) methanol solution, then 0.04% ascorbic solution was added as an antioxidant. After that, 3 drops of 1.2 M of HCl solution were added, and then the resultant was refluxed at 80 °C for 2 h. The resultant was allowed to cool after the completion of the hydrolysis process and shifted to a volumetric flask, and then volume was made up to 10 mL with methanol and filtered by 0.45 µm non-pyrogenic filters before being subjected to injection.

#### 2.8.2. Preparation of Calibration Curves

Pure standards were dissolved in analytical grade methanol to form 1000 µg/mL solution. MeOH (concentration of 0.4–400 µg/mL) was needed to prepare the standard solutions and each standard was obtained by using this calibration curve.

#### 2.8.3. Chromatographic Conditions

The separation and quantification of phenolic acids and flavonoids were performed by HPLC system (Agilent Technologies, Inc., SantaClara, CA, USA) as reported previously [24]. Data analysis was performed using software, version 4.2. 6410. Matching of retention time and spiking techniques were used for qualitative analysis, and quantification was performed using the external standard calibration curve method.

### 2.9. Evaluation of Antimicrobial Activity

#### 2.9.1. Microbial Culture and Growth Conditions

Both Gram-positive and Gram-negative multidrug-resistant isolates of *Staphylococcus aureus* (three isolates), methicillin-resistant *Staphylococcus aureus* (MRSA), *Escherichia coli* (three isolates), *Klebsiella* sp. (two isolates), *Pseudomonas aeruginosa,* and *Acinetobacter* species were provided by Department of Microbiology, Government College, University Faisalabad, Pakistan. For quality control, standard strains such as *S. aureus* ATCC 25923 and *E. coli* ATCC 25922 were used. For the conservation of these selected clinical isolates, nutrient agar slants were employed at 4 °C [27]. Twenty-four hours before testing, the cultures were sub-cultured on nutrient broth. For the antimicrobial assay, these selected isolates serving as test pathogens were used.

#### 2.9.2. Susceptibility Test

The antimicrobial activity of all the tomato fruit extracts was analyzed using agar well diffusion assay [1].

#### 2.9.3. Minimum Inhibitory Concentration (MIC)

On the basis of preliminary results, the extracts showed that maximum inhibition was further tested for their MIC against the three isolates, *S. aureus* isolate 1, *S. aureus* isolate 3, and *S. aureus* MRSA. The MIC value of the tomato fruit extracts grown in T1, T2, and T3 soils was determined using the resazurin indicator as suggested by Riss et al. [28]. A stock solution of 0.015% (*w/v*) resazurin indicator in DPBS (pH 7.4) was filter-sterilized and stored in a sterile light protectant container at 4 °C. The bacterial strains (100 μL/well), the stock solution of extracts (in a concentration ranging from 0.1–18 (μL/well), nutrient broth, and 10% methanol (20 μL/well) were added to each well of the 96-well plate to make a final volume of 300 μL in each well. Moreover, the 96-well plate was kept for 24 h at 37 °C and then 30 μL of resazurin indicator was added to each well. The plate was incubated again for 2–4 h at 37 °C to observe the color change. Post-incubation, MIC values were recorded for wells where no color change was observed.

#### 2.9.4. Minimum Bacterial Concentration (MBC)

The minimum bactericidal concentration (MBC) for each tomato extract (T1, T2, and T3) was ascertained by directly plating the content of wells from the 96-well plate which did not show any visible sign of growth onto sterile nutrient agar plates. The dilution that yielded no colony growth was noted as MBC [1].

### 2.10. Statistical Analysis

Three samples of tomatoes were collected and analyzed in triplicate and the values were expressed as mean ± SD. The significant differences among the numerical values were analyzed using one-way analysis of variance (ANOVA) followed by Tukey’s test, using Minitab version 18. The level of significance was set at *p* ≤ 0.05.

## 3. Results and Discussion

### 3.1. Proximate Analysis and Vitamin C Contents

Proximate analysis of tomatoes grown in T1 (soil 100% + 0% fly ash), T2 (soil 75% + 25% fly ash), and T3 (soil 50% + 50% fly ash) soils is presented in Table 1**.** The moisture contents of tomatoes decreased as the concentration of fly ash increased from 93.05 to 86.97 g/100 g. Moreover, ash contents of tomatoes increased as the concentration of fly ash increased, with order T1 < T2 < T3, i.e., 4.81, 5.33, 5.95 g/100 g, respectively. Experimental data regarding the moisture and ash contents of these observable tomato varieties were very close to the results reported in the literature [29].

The results regarding the crude fat and crude fiber contents of tomatoes grown in fly ash blended soil, such as T1, T2, and T3 soils, are reported in Table 1. The mean percentages of crude fat of tomatoes grown in T3 soil were found to be higher than tomatoes grown in other soils. Similarly, the crude fiber of tomatoes was found to be 1.42, 1.99, and 2.34 g/100 g for T1, T2, and T3 soils, respectively. Moreover, the mean percentages of total carbohydrate of tomatoes grown in different soils were found in the following order: T3 > T2 > T1. Crude fiber and crude fat are effective nutrients helpful in stool weight to increase and benefit alimentary canal by gastrointestinal transit time reduction [30]. For the proper growth and body maintenance, protein plays a key role and, along with lipids and carbohydrates, acts as an energy source [31]. The crude proteins of tomatoes grown in T1, T2, and T3 soils were found to be 9.66, 10.37, and 12.38 g/100 g, respectively. Significance (*p* ≤ 0.05) variations were observed in moisture, ash, crude fat, crude fiber, total carbohydrate, and crude protein of tomatoes grown in different fly ash blended soils. Comparable results were observed as compared to the proximate analysis of different varieties of tomato [29,30].

Data recording vitamin C of tomatoes grown in different fly ash blended soils are given in Table 1. Vitamin C contents of tomatoes grown in T1, T2, and T3 soils were found to be 13.55, 13.89, and 14.44 mg/100 g, respectively. Tomatoes grown in T3 soil contained the highest vitamin C contents, followed by T2 and T1 soils. Tomatoes grown in different fly ash blended soils showed significant (*p* ≤ 0.05) variations in the vitamin C contents. Results are comparable with previously reported data on tomato genotypes [31,32].

### 3.2. Antioxidant Assay

In this study, the tomato fruit extracts obtained from T3 soil exhibited the highest TPC (45.59 mg/100 g dry weight, measured as GAE) and TFC (15.98 mg/100 g dry weight, measured as CE), followed by T2 soil TPC (29.52 mg/100 g, GAE) and TFC (5.01 mg/100 g, CE) and T1 soil TPC (23.42 mg/100 g, GAE) and TFC (2.18 mg/100 g, CE) as shown in Table 1. Moreover, tomato extract of T3 soil showed maximum DPPH free radical scavenging activity with the lowest IC_50_ value (24.52 μg/mL), followed by T2 soil (33.63 μg/mL) and T1 soil (40.71 μg/mL), as shown in Table 1. In the FRAP assay, the antioxidant potential of the tomato extracts can be estimated by its reducibility of Fe^3+^ to Fe^2+^ at a lower pH leading to the formation of an intense blue-colored due to formation of ferrous tripyridyltriazine complex (Fe^2+^-TPTZ) [25]. FRAP values of 179.28, 193.01, and 220.23 mmol/L ferrous equivalents were observed for tomato extract of T1, T2, and T3 soils, respectively. Some reports in literature also reported higher antioxidant activity in beetroot, rice, and chickpea that were grown in soil treated with different concentration of fly ash [33,34,35]. Higher antioxidant potential with significant (*p* < 0.05) variation of all observed blended soils fruits can be justified due to presence of high concentration of phenolics and flavonoids.

### 3.3. Element Analysis

Elements analysis of tomatoes grown in T1, T2, and T3 soils was performed, and results are reported in Table 2. Tomatoes grown in T3 soils were found to have higher concentration of Ca (1037.2 mg/kg), Cu (0.93 mg/kg), Fe (6.44 mg/kg), Mn (1.87 mg/kg), and Ni (98.6 mg/kg), and lower concentration of Zn (0.40 mg/kg), Mg (40.8 mg/kg), Pb (0.37 mg/kg), and Cd (181.09 mg/kg) as compared with T2 and T1 soils. Most importantly, concentration of Pb and Cd decreases in T2 and T3 blended soils as the concentration of fly ash is increased, whereas the rest of the heavy metals showed increasing order. All the observed tomatoes grown in different fly ash blended soils showed significant (*p* < 0.05) variation for element analysis. Our findings showed results which are very similar with the reported findings by Jamil et al. [36] and Chhabra et al. [1], where heavy metal uptake was increased in roots, stem, leaves, and fruits, respectively, after the amendments of soil with different concentration of fly ash.

### 3.4. Phenolic and Flavonoids Profile Analysis by HPLC

**Figure 1:**
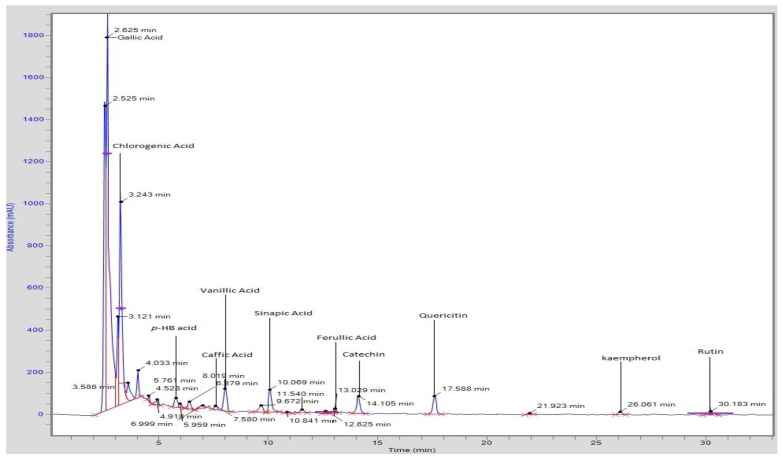
Typical HPLC chromatogram of phenolic acid and flavonoids.

The HPLC analysis revealed that eight phenolic and four flavonoids were identified for tomato grown in T1, T2, and T3 soils (Table 3, Figure 1). The higher concentration of major phenolics detected were gallic acid (300.2 mg/100 g of dry plant material), followed by chlorogenic acid (93.45 mg/100 g), *p*-coumeric acid (67.29 mg/100 g of dry plant material), vanillic acid (27.89 mg/100 g), hydroxybenzoic acid (24.27 mg/100 g), sinapic acid (8.29 mg/100 g of dry plant material), caffeic acid (15.36 mg/100 g of dry plant material), and ferulic acid (17.99 mg/100 g). Some other major flavonoids were kaempferol (1233.7 mg/100 g), followed by catechin (239.2 mg/100 g), rutin (129.6 mg/100 g), and quercetin (8.45 mg/100 g) in tomato fruit extract of T3 soil, as compared with T2 and T1 soils. Moreover, the phenolics and flavonoids contents of tomatoes grown in different soils were found in the following order: T3 > T2 > T1. Significant (*p* ≤ 0.05) variations were observed in phenolic acids and flavonoids of tomatoes grown in different fly ash blended soil. Our findings regarding the phenolic profile and flavonoids are in agreement with the findings previously reported by Silva-Beltrán et al. [37] in tomato fruit extract which exposed the presence of gallic acid, caffeic acid, chlorogenic acid, ferulic acid, quercetin, and rutin in the forensic study.

### 3.5. Antibacterial Activity

Antibacterial activity of tomatoes, grown in different fly ash blended soils (T1, T2, and T3) were performed and results are presented in Table 4. No zone of inhibition was observed for the Gram-negative isolates tested except *E. coli*. Inhibition zones of samples against clinical isolates ranged from 10–15 mm in tested Gram-positive isolates and 8.5–11.5 mm in the case of Gram negative bacteria. Tomatoes grown in T3 soil exhibited maximum inhibition zone of 15.0, 14.7, and 14.5 mm in diameter against Gram-positive bacteria (*Staphylococcus aureus*1, *Staphylococcus aureus* MRSA, and *Staphylococcus aureus*3) followed by T2 and T1 soil. This higher antimicrobial activity against Gram-positive bacteria and lower against Gram-negative bacteria can be explained by the difference in the cell wall structure of both types of bacteria. The cell wall of Gram-negative bacteria is composed of lipopolysaccharides and phospholipids that act as an impediment which protects it from different actions of phytochemicals [38]. All the fly ash blended soils tomato, T1, T2, and T3, were observed to be virtually inactive against *Acinetobacter* sp., *E*. *coli* isolate 3, and *Klebsiella* sp. isolate 1, 2. All these isolates possessed resistance towards the majority of antibiotics tested [26]. The results obtained in this study are similar with the findings of previously reported data [39]. The tomato fruit extracts of T1, T2, and T3 possessed significant inhibition (*p* < 0.05) towards the growth of both Gram-positive and Gram-negative bacteria. This increase in antibacterial activity may be due to the high elements concentration and higher production of phenolics and flavonoids in T3 soil tomatoes. Reactive oxygen species (ROS) can be reduced by heavy metals present in fly ash and absorbed by plants [40].

The MIC and MCB values of tomato extracts for the tested organisms ranged between 4.00–10.0 mg/mL and 3.25–12.0 mg/mL in all the fly ash blended T1, T2, and T3 soils and are presented in Table 5. Tomatoes grown in T3 soil were observed to be the most active against *S.aureus*1 and *S. aureus*3 (MIC value 4.00 mg/mL) followed by T2 soil tomatoes (MIC value 5.25 mg/mL). In T2 and T3, soil tomatoes’ maximum MIC value of 7.5 mg/mL was reported against *S. aureus* MRSA. Identical values were observed by Chhabra et al. [1], Rampadarath et al. [41], and Sharma et al. [42] when tested against *S. aureus* isolates.

## 4. Conclusions

Fruit extracts obtained from tomatoes grown in soil blended with different concentrations of fly ash, i.e., T1 (soil 100% + 0% fly ash), T2 (soil 75% + 25% fly ash), and T3 (soil 50% + 50% fly ash), exhibited significant variation in the antioxidant and antimicrobial activities against various clinical multidrug-resistant isolates. Generally, T3 soil crop showed best antioxidant and antimicrobial potentials. HPLC results also confirmed increase of phenolic acids and flavonoids in T3 soil tomatoes followed by T2 soil tomatoes, when T1 soil tomatoes were taken as reference. Moreover, soil with high concentration of fly ash (T3 soil) showed impacts on the TPC and TFC of tomatoes, as well as on the various other parameters, such as proximate composition, vitamin C, and mineral contents. All the above results are supported by previous work on beetroot [43] and *Jatropha curcas* [1]. Fly ash, when used in synergy with soil at sustainable amounts, can prompt the production of beneficial bioactive components. Thus, ameliorating the soil with fly ash can serve as an ecofriendly and cost-effective method for increasing the medicinal potential of the plant by increasing elemental concentration and phenolics contents which may improve its bioactivity, consequently increasing the therapeutic potential of the plant.

## Figures and Tables

**Table 1:** Proximate composition, vitamin C content, and antioxidant activity of tomato grown in different blended soils.

Sr. No	Compounds	T1	T2	T3	BHT
1	Moisture (g/100 g)	93.05 ± 3.6 ^a^	90.50 ± 2.7 ^a^	86.97 ± 2.6 ^b^	---
2	Ash (g/100 g)	4.81 ± 0.04 ^c^	5.33 ± 0.04 ^b^	5.95 ± 0.05 ^a^	---
3	Crude fat (g/100 g dry fruit)	0.57 ± 0.02 ^c^	0.78 ± 0.02 ^b^	1.06 ± 0.03 ^a^	---
4	Crude fiber (g/100 g dry fruit)	1.42 ± 0.07 ^c^	1.99 ± 0.06 ^b^	2.34 ± 0.06 ^a^	---
5	Total Carbohydrate (g/100 g dry fruit)	1.75 ± 0.08 ^c^	1.95 ± 0.07 ^b^	2.16 ± 0.09 ^a^	---
6	Crude protein (g/100 g dry fruit)	9.66 ± 0.40 ^c^	10.37 ± 0.4 ^b^	12.38 ± 0.4 ^a^	---
7	Vitamin C (mg/100 g Fw)	13.55 ± 0.5 ^c^	13.89 ± 0.4 ^b^	14.44 ± 0.5 ^a^	---
8	TPC	23.42 ± 1.17 ^c^	29.52 ± 1.42 ^b^	45.59 ± 2.07 ^a^	---
9	TFC	2.18 ± 0.01 ^c^	5.01 ± 0.10 ^b^	15.93 ± 0.34 ^a^	---
10	IC_50_ value (μg/mL)	40.71 ± 0.04 ^a^	33.63 ± 0.03 ^b^	24.52 ± 0.02 ^c^	8.12 ± 003 ^d^
11	FRAP (mmol/L)	179.13 ± 2.66 ^c^	193.01 ± 2.14 ^b^	220.23 ± 1.83 ^a^	---

Values are mean ± SD in triplicate determinations. Different letters in superscript represent significant (*p* ≤ 0.05) difference among tomatoes grown in T1 (soil 100% + 0% fly ash), T2 (soil 75% + 25% fly ash), and T3 (soil 50% + 50% fly ash) soils. TPC: Total phenolic content measured as mg/g of dry plant material, as compared to gallic acid equivalent. TFC: Total flavonoids content measured as mg/g of dry plant material, as compared to catechin equivalent. IC_50_: DPPH radical scavenging activity in term of IC_50_.

**Table 2:** Element analysis of tomato fruits grown in different fly ash blended soils.

Blending	Heavy Metals (mg/kg)								
	Zn	Ca	Mg	Pb	Cu	Fe	Mn	Ni	Cd
T1	1.34 ± 0.02 ^a^	926.3 ± 18.5 ^c^	52.1 ± 2.08 ^a^	0.48 ± 0.01 ^a^	0.36 ± 0.01 ^c^	3.89 ± 0.13 ^c^	1.06 ± 0.03 ^c^	37.66 ± 0.73 ^c^	242.05 ± 4.45 ^a^
T2	0.92 ± 0.04 ^b^	987.1 ± 18.9 ^b^	49.9 ± 2.50 ^b^	0.46 ± 0.00 ^b^	0.42 ± 0.01 ^b^	4.37 ± 0.21 ^b^	1.42 ± 0.02 ^b^	42.50 ± 1.34 ^b^	233.67 ± 6.76 ^b^
T3	0.40 ± 0.02 ^c^	1037.2 ± 20.7 ^a^	40.8 ± 1.63 ^c^	0.31 ± 0.01 ^c^	0.93 ± 0.04 ^a^	7.44 ± 0.25 ^a^	1.87 ± 0.03 ^a^	158.6 ± 3.16 ^a^	181.09 ± 3.58 ^c^

Values are mean ± SD in triplicate determinations. Different letters in superscript represent significant (*p* ≤ 0.05) difference among tomatoes grown in T1 (soil 100% + 0% fly ash), T2 (soil 75% + 25% fly ash), and T3 (soil 50% + 50% fly ash) soils. Zn = zinc, Ca = calcium, Mg = magnesium, Pb = lead, Cu = copper, Fe = iron, Mn = manganese, Ni = nickel, Cd = cadmium.

**Table 3:** Phenolic acids and flavonoids contents by HPLC.

Sr. No	Compounds	T1	T2	T3
1	Gallic acid	272.2 ± 8.1 ^c^	293.5 ± 8.3 ^b^	300.2 ± 8.20 ^a^
2	Chlorogenic acid	75.60 ± 2.5 ^c^	79.2 ± 2.56 ^b^	93.45 ± 2.58 ^a^
3	Hydroxybenzoic acid	13.30 ± 0.51 ^c^	18.9 ± 0.48 ^b^	24.27 ± 0.49 ^a^
4	Caffeic acid	5.220 ± 0.22 ^c^	9.80 ± 0.24 ^b^	15.36 ± 0.23 ^a^
5	Vanillic acid	16.50 ± 0.91 ^c^	19.6 ± 0.91 ^b^	27.89 ± 0.92 ^a^
6	*p*-coumeric acid	-	52.8 ± 1.32 ^b^	67.29 ± 1.38 ^a^
7	Sinapic acid	5.900 ± 0.24 ^c^	7.01 ± 0.21 ^b^	8.290 ± 0.22 ^a^
8	Ferulic acid	3.720 ± 0.01 ^c^	9.80 ± 0.02 ^b^	17.99 ± 0.02 ^a^
9	Catechin	213.9 ± 7.41 ^c^	228.2 ± 7.40 ^b^	239.2 ± 7.03 ^a^
10	Quercetin	7.700 ± 0.42 ^c^	7.90 ± 0.41 ^b^	8.450 ± 0.41 ^a^
11	Kaempferol	1181.7 ± 35.40 ^c^	1198.5 ± 31.20 ^b^	1233.7 ± 30.17 ^a^
12	Rutin	107.6 ± 4.34 ^c^	115.3 ± 4.22 ^b^	129.6 ± 4.27 ^a^

Values are mean ± SD in triplicate determinations. Different letters in superscript represent significant (*p* ≤ 0.05) difference among tomatoes grown in T1 (soil 100% + 0% fly ash), T2 (soil 75% + 25% fly ash), and T3 (soil 50% + 50% fly ash) soils. (-): Not present.

**Table 4:** Antibacterial activity of tomato samples T1, T2, and T3 by agar well diffusion method.

Sr. No	Test Organisms	T1	T2	T3
1	*Staphylococcus aureus*1	10.0 ± 0.60 ^c^	12.0 ± 0.52 ^b^	13.0 ± 0.65 ^a^
2	*Staphylococcus aureus*2	11.0 ± 0.32 ^c^	13.0 ± 0.47 ^b^	14.0 ± 0.42 ^a^
3	*Staphylococcus aureus*3	12.0 ± 0.47 ^c^	13.0 ± 0.55 ^b^	14.0 ± 0.58 ^a^
4	*Staphylococcus aureus* MRSA	11.0 ± 0.33 ^c^	12.0 ± 0.63 ^b^	13.7 ± 0.53 ^a^
5	*Staphylococcus aureus* ATCC 25923	13.0 ± 0.35 ^c^	14.5 ± 0.45 ^b^	14.4 ± 0.47 ^a^
6	*Pseudomonas aeruginosa*	-	-	-
7	*Acinetobacter* sp.	-	-	-
8	*Escherichia coli* 1	8.5 ± 0.19 ^c^	9.4 ± 0.14 ^b^	10.0 ± 0.23 ^a^
9	*Escherichia coli* 2	9.0 ± 0.22 ^b^	9.0 ± 0.15 ^b^	9.50 ± 0.18 ^a^
10	*Escherichia coli* 3	-	-	-
11	*Escherichia coli* ATCC 25922	9.2 ± 0.30 ^b^	10.0 ± 0.27 ^b^	11.5 ± 0.16 ^a^
12	*Klebsiella* sp.1	-	-	-
13	*Klebsiella* sp.1	-	-	-

Values are mean ± SD in triplicate determinations. Different letters in superscript represent significant (*p* ≤ 0.05) difference among tomatoes grown in T1 (soil 100% + 0% fly ash), T2 (soil 75% + 25% fly ash), and T3 (soil 50% + 50% fly ash) soils. (-): Not present.

**Table 5:** MIC and MBC values (mg/mL) of tomato fruit extracts (samples T1, T2, and T3) against different bacterial species.

Test Organism	*S. aureus*1		*S. aureus*3		*S. aureus* MRSA	
Extract	MIC	MBC	MIC	MBC	MIC	MBC
T1	5.00 ± 0.15 ^c^	6.5 ± 0.16 ^b^	6.50 ± 0.17 ^b^	12.00 ± 0.35 ^a^	8.0 ± 0.18 ^a^	12.0 ± 0.17 ^a^
T2	2.25 ± 0.06 ^b^	3.5 ± 0.07 ^c^	2.25 ± 0.05 ^b^	6.50 ± 0.16 ^b^	10.0 ± 0.19 ^a^	12.0 ± 0.16 ^a^
T3	5.00 ± 0.13 ^b^	6.5 ± 0.14 ^b^	5.00 ± 0.12 ^b^	2.25 ± 0.05 ^c^	10.0 ± 0.17 ^a^	10.0 ± 0.17 ^a^

Values are mean ± SD in triplicate determinations. Different letters in superscript represent significant (*p* ≤ 0.05) difference among tomatoes grown in T1 (soil 100% + 0% fly ash), T2 (soil 75% + 25% fly ash), and T3 (soil 50% + 50% fly ash) soils. *S. aureus*: *Staphylococcus aureus*.

## Data Availability

The data is contained within the article.
